# A205 PRACTICE PATTERNS FOR EOSINOPHILIC ESOPHAGITIS IN ADULTS VARY WIDELY AMONG CANADIAN GASTROENTEROLOGISTS: INTERIM ANALYSIS OF A NATIONWIDE SURVEY

**DOI:** 10.1093/jcag/gwad061.205

**Published:** 2024-02-14

**Authors:** A Fetz, H Kim, S Moosavi

**Affiliations:** The University of British Columbia, Vancouver, BC, Canada; The University of British Columbia, Vancouver, BC, Canada; The University of British Columbia, Vancouver, BC, Canada

## Abstract

**Background:**

Eosinophilic esophagitis (EoE) is a chronic allergic, type 2 immune-mediated, condition of the esophagus with increasing prevalence in Canada. Practice patterns among Canadian gastroenterologists are not well described.

**Aims:**

To identify practice variation among Canadian gastroenterologists who care for adults with EoE.

**Methods:**

A cross-sectional, web-based survey was distributed to Canadian gastroenterologists through the Canadian Association of Gastroenterology as well as administrations at Canadian universities.

**Results:**

Of 53 respondents, 68% currently work in an academic setting. 77% manage at least 6 patients with EoE per year, with 19% managing over 50 patients per year.

51% of respondents obtain biopsies in every new patient presenting with an acute food bolus, while 26% take biopsies in less than 50% of cases or never at all. 36% take less than 5 biopsies on initial endoscopy with 23% only taking biopsies from one location in the esophagus. 58% are either unaware of the EoE Endoscopic Reference Score (EREFS) or never use it in clinical practice.

Most respondents (72%) prefer proton pump inhibitors as initial therapy while 17% prefer starting multiple treatment modalities. 9% use topical steroids initially. Only 6% keep their patients on topical steroids indefinitely, if started. Preferred dietary approach vary with 36% choosing 6-food elimination diet (FED), 23% 2-FED, 19% 4-FED, 13% milk-only elimination diet, 6% allergy test-directed diet, and 4% elemental diet. Only 17% feel very comfortable starting biologic therapies.

When evaluating treatment effectiveness, 23% use improving patient symptoms alone, while 74% include repeat histological assessments. 42% do not regularly screen for adaptive behaviours, such as excessive chewing, or texture modification and avoidance. In those who perform repeat endoscopic evaluation, 13% do so in less than 3 months after starting therapy, 32% in 3-5.9 months, 28% in 6-11.9 months, and 8% after a year. 11% will only repeat endoscopic evaluation if a patient reports symptom recurrence. 64% do not regularly refer to an allergist.

For guidance, 59% follow the American Gastroenterology Association 2020 guidelines, 34% American Society of Gastrointestinal Endoscopy 2022 guidelines, 4% British Society of Gastroenterology 2022 guidelines, while 21% do not use any guidelines. 83% feel Canadian guidelines would be useful in their practice.

**Conclusions:**

EoE practice patterns among Canadian adult gastroenterologists vary widely, with management differing from the current consensus guidelines. Strategies aimed at decreasing these variations, such as the development of Canadian guidelines, are needed to standardize practice.

**Funding Agencies:**

None

